# Antibiotics Resistance and Biofilm Formation Capacity of *Staphylococcus* spp. Strains Isolated from Surfaces and Medicotechnical Materials

**DOI:** 10.1155/2020/6512106

**Published:** 2020-08-27

**Authors:** Akim Socohou, Haziz Sina, Cyriaque Degbey, Chimène Nanoukon, Kamirou Chabi-Sika, Hélène Ahouandjinou, Halfane Lehmane, Farid Baba-Moussa, Lamine Baba-Moussa

**Affiliations:** ^1^Laboratoire de Biologie et de Typage Moléculaire en Microbiologie, Département de Biochimie et de Biologie Cellulaire, Faculté des Sciences et Techniques, Université d'Abomey-Calavi, Cotonou 05 BP 1604, Benin; ^2^Institut Régional de Santé Publique de Ouidah, Université d'Abomey-Calavi, Ouidah BP 384, Benin; ^3^Laboratoire de Microbiologie et des Technologies Alimentaires, Département de Biologie Végétale, Faculté des Sciences et Techniques, Université d'Abomey-Calavi, Cotonou 01 BP 526, Benin

## Abstract

*Staphylococcus* spp. is most often implicated in nosocomial infections. The objective of this study is to evaluate the susceptibility to antibiotics and the biofilm formation capacity of staphylococci species isolated from surfaces and medicotechnical materials at the university hospital center of Abomey-Calavi/Sô-Ava in Benin. Samples were collected according to ISO/DIS14698-1 standard from the surfaces and medicotechnical materials by the dry swab method. The isolation of *Staphylococcus* strains was performed on Chapman agar, and their identification was performed using microscopic and biochemical methods. The susceptibility of *Staphylococcus* isolates to antibiotics was evaluated by the disc diffusion method according to EUCAST and CLSI recommendations. The biofilm formation was qualitatively assessed using microplates. Of the 128 surfaces and medicotechnical material samples analyzed, 77% were contaminated with *Staphylococcus* spp. Thirteen species of *Staphylococcus* were isolated in different proportions but the pediatric department was the most contaminated (33%) by *S*. *aureus*. Resistance to antibiotics considerably varies according to the species of *Staphylococcus*. However, antibiotics such as chloramphenicol and vancomycin are the most effective on *S*. *aureus*, whereas coagulase-negative staphylococci developed less resistance to gentamycin and ciprofloxacin. The biofilm test reveals that 37% of our isolated strains were biofilm formers. Although regular monitoring of hospital hygiene is crucial, the optimal use of antibiotics is a cornerstone of reducing antimicrobial resistance.

## 1. Introduction

The hospital is a place where the risk of infection is very high. These hospital-acquired (nosocomial) infections are recognized as a real public health problem because of their frequency, socioeconomic cost, and severity. Those infections affect patients, their families, and all health professionals. The causes of nosocomial infections are multiple, linked both to care procedures and to behavioral practices. Several studies show that *Escherichia coli* and *Staphylococcus aureus* are predominantly isolated from all nosocomial infections [1]. In a study conducted by Ahoyo et al. [2] in the pediatric unit of the Zou and Collines departmental hospital (Benin), 32% of nosocomial infection involving *S*. *aureus* was reported in the care environment.

In fact, staphylococci were identified at the dawn of the Pasteur era and have never ceased to give rise to research, as their importance is so great in pathology. They occupy a significant proportion among the bacteria responsible for serious infections. Moreover, they are observed in multiple clinical situations, both in community and nosocomial pathologies [3]. In addition, staphylococci are predominant pathogens of postoperative infections. Among these, coagulase-negative *Staphylococcus* are the main agents on materials [4]. Some *Staphylococcus* species can also survive on inanimate surfaces such as bedding, clothing, and door handles [5]. This general tendency to adhere to various surfaces is produced by a polysaccharide matrix called biofilm, and this factor confers significant resistance to antibiotics and to attacks by the immune system [6, 7]. In hospitals, the selection pressure exerted by antibiotics and antiseptics reinforces the emergence of the most resistant bacteria. Thus, hospital environment appears like multidrug-resistant bacteria reservoirs. This is combined with the many risk factors for cross-transmission of pathogenic germs and can explain their involvement in nosocomial infections [8]. Moreover, mortality linked to infections with multidrug-resistant bacteria remains very high worldwide [9]. Several cases of multidrug-resistant bacteria are reported in Benin [10] and in other sub-Saharan African countries [11].

The predominance of *Staphylococcus* spp. in hospitals indicates a noncompliance with hygiene rules [12, 13]. The present study was conducted for a better knowledge of pathogenic *Staphylococcus* strains for an effective therapeutic approach and a better use of antibiotics. Thus, our study aims at drawing up the resistance profile of *Staphylococcus* species isolated from the Abomey-Calavi/Sô-Ava university hospital center and determining their capacity to form bacterial biofilm.

## 2. Material and Methods

### 2.1. Sampling

The samples were collected in the university center hospital of Abomey-Calavi/Sô-Ava (Southern Benin) from January to June 2019 in 5 departments (Neonatology, Pediatrics, Maternity, Operating room, and Central sterilization) according to ISO/DIS14698-1 [14]. For the study, 128 samples were collected by the dry swab method from surfaces and medicotechnical materials such as beds, soils, carriages, baby vanity tables, weighs baby, mattresses, cupboard, and Caesarean boxes. For the sampling, after passing the swabs over defined areas, they were returned to their protective cases. The collected samples were transported using icebox containing coolers (∼8°C) and then 5 ml of Mueller Hinton broth was added to each case and then incubated at 37°C for 24 h. Three repetitions were done for each surface and equipment.

### 2.2. Isolation and Identification of Isolated Strains

The isolation of *Staphylococcus* bacteria was carried out on Chapman agar. In brief, after 24 hours of incubation, the cases having the cloudy appearance testifying to a bacterial growth were incubated at 37°C on Chapman agar for 24 hours [15]. The identification of *Staphylococcus* strains was carried out using microscopic and biochemical methods (Gram stain, DNase test, and catalase test) and API® Staph (bioMerieux, France).

### 2.3. Susceptibility of Strains to Antibiotics

The susceptibility of *Staphylococcus* strain isolated to 15 antibiotics was investigated by the disc diffusion method on the Mueller Hinton agar medium according to the EUCAST [16] and CLSI [17] recommendations. The bacterial suspension was standardized using the 0.5 McFarland control. Fifteen tested antibiotics were penicillin G (P 10 *μ*g), vancomycin (VA 30 *μ*g), fosfomycin (FOS 50 *μ*g), tetracycline (OT 30 *μ*g), amoxiclav (AC 30 *μ*g), cefoxitin (FOX 30 *μ*g), gentamycin (G 10 *μ*g), (C 30 *μ*g), cephalothin (KC 30 *μ*g), kanamycin (K 30 *μ*g), erythromycin (E 15 *μ*g), ciprofloxacin (CF 5 *μ*g), streptomycin (S 10 *μ*g), trimethoprim (TMP 5 *μ*g), chloramphenicol (C 30 *μ*g), and ceftriaxone (CI 30 *μ*g).

### 2.4. Bacterial Biofilm Formation Test

The bacterial capacity to form biofilm was determined using the method previously described by the Christensen et al. [18]. Therefore, we used *in vitro* microplate study models to assess qualitatively biofilm formation because of the occurrence of visible film. Thus, from an 18 h culture in Brain Heart Infusion (BHI) Broth medium, a 48-well microplate was inoculated with 10 *μ*l of bacteria suspension to which 150 *μ*l of BHI was added. The microplates were incubated for 24 hours at 37°C and wells were washed three times with 0.2 ml of sterile physiological water in order to eliminate the free bacteria. The biofilms formed by the adhesion of sessile organisms to the polystyrene support in each of the wells were stained with violet crystal (0.1%) for 10 min. The excess dye was then removed by thorough washing with sterile distilled water and the plates were left at room temperature for drying [19]. After air-drying, the occurrence of visible film lined the microplate walls, and the bottom of the walls indicates biofilm production.

### 2.5. Data Analysis

Data were recorded and analyzed with MS Excel 2013 Spreadsheet. The percentage of resistance was calculated for each antibiotic by dividing the frequency of resistant bacteria by the number of bacteria tested. The Graph Pad Prism 7.00 software was used for the graphs. The threshold of statistical significance was set at *p* < 0.05.

## 3. Results

### 3.1. Identification of Bacteria

**Figure 1:**
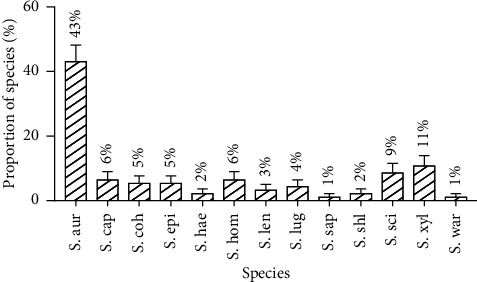
Global distribution of isolated *Staphylococcus* strains.

Among the 128 samples collected in the study, 77% were contaminated with *Staphylococcus* spp., spread in different proportions, into 13 species, namely: *S*. *aureus*, *S*. *capitis*, *S*. *cohnii* ssp. *cohnii*, *S*. *epidermidis*, *S*. *haemolyticus*, *S*. *hominis*, *S*. *lentus*, *S*. *lugdunensis*, *S*. *saprophyticus*, *S*. *schleiferi*, *S*. *sciuri*, *S*. *xylosus*, and *S*. *warneri*. Thus, independent of the unit of sample collection, *Staphylococcus aureus* was the most predominant (43%) followed by *S*. *xylosus* (11%). *S*. *saprophyticus* and *S*. *warneri* (1%) were the least isolated (Figure 1).

**Figure 2:**
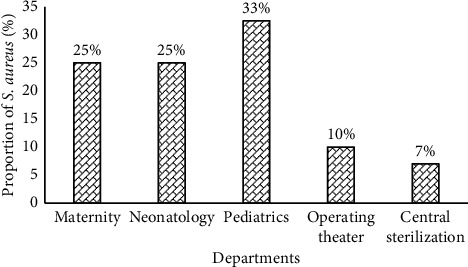
Distribution by department of the *Staphylococcus aureus* strains isolated.

The distribution of the major species is very variable depending on the sampling units. It thus appears that the pediatric unit is the most contaminated (33%) by the strains of *S*. *aureus* followed by maternity and neonatology (25%), and the central sterilization unit is the least contaminated (7%) (Figure 2).

### 3.2. Susceptibility to Antibiotics

The isolated strains were split into two categories for the assessment of susceptibility to antibiotics. *S*. *aureus* is the coagulase-positive staphylococci (CPS) isolated and the other 12 species (*S*. *capitis*, *S*. *cohnii* ssp. *cohnii*, *S*. *epidermidis*, *S*. *haemolyticus*, *S*. *hominis*, *S*. *lentus*, *S*. *lugdunensis*, *S*. *saprophyticus*, *S*. *schleiferi*, *S*. *sciuri*, *S*. *xylosus*, and *S*. *warneri*) are coagulase-negative staphylococci (CNS). Thus, it is observed that all of the *S*. *aureus* strains are resistant to cephalothin followed by resistance level to fosfomycin (92.5%) and cefoxitin (87.5%). The lowest resistance of *S*. *aureus* was recorded with chloramphenicol (15%) and vancomycin (25%).

Considering the coagulase-negative staphylococci, there was recorded high resistance to fosfomycin (94%) and penicillin (87%). The lowest resistance in CNS was observed with gentamycin (17%) and ciprofloxacin (17%) (Table 1).

### 3.3. Biofilm Research Test

**Figure 3:**
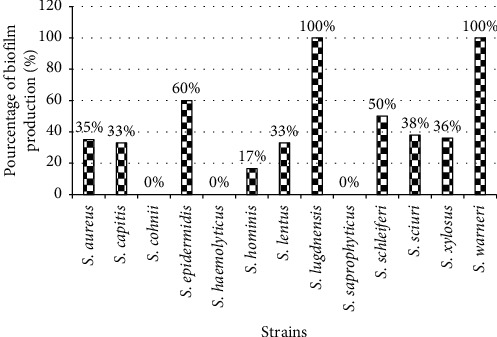
Profile of biofilm formation by isolated *Staphylococcus* strains.

The biofilm formation test reveals that 37% of our isolates were biofilm formers. When considering species, we observe that 100% of the species of *S*. *lugdunensis* and *S*. *warneri* isolated were biofilm-forming bacteria followed by *S*. *epidermidis* (60%). However, no biofilm formation was noticed with species such as *S*. *cohnii* ssp. *cohnii*, *S*. *haemolyticus*, and *S*. *saprophyticu*s (Figure 3) isolated in our study.

## 4. Discussion

Among the thirteen identified staphylococci species, there was a predominance of *Staphylococcus aureus* (43%). A high proportion (∼45%) of *Staphylococcus aureus* from hospital environment samples has been reported in Benin [13] and Morocco [20]. However, in Mali, *S*. *epidermidis* was reported to be the predominant species in the hospitals [21]. The frequency and the rate of isolated species vary according to their sampling site. Thus, it can be mentioned that species exclusively from human origin (*S*. *capitis*, *S*. *hominis*, *S*. *lugdunensis*, and *S*. *schleiferi*), species of both human and animal origin (*S*. *aureus*, *S*. *cohnii*, *S*. *haemolyticus*, *S*. *warneri*, and *S*. *xylosus*), and species of animal origin (*S*. *hyicus*, *S*. *lentus*, and *S*. *sciuri*) were observed [21]. The presence of those species (animal and/or human) in the hospital environment is evidence of human contamination and suggests contact between patients and animals or between health personnel and animals in their living environment. *S*. *aureus* is the unique coagulase-positive strain isolated, and its pathogenicity is reported to be related to the expression of several virulence factors [22]. In addition, some CNS such as *S*. *saprophyticus* and *S*. *epidermidis* through their ability to adhere to the bladder epithelium are able to cause cystitis in young women, and *S*. *lugdunensis* is responsible for skin infections and infectious endocarditis [23].

The distribution of species according to the sampling units shows that *S*. *aureus* isolates are found in all the units. However, pediatrics unit was the most contaminated (33%) by *S*. *aureus*. This high presence in these various units is worrying when we know that the deficient immune status of patients represents a breeding ground for its pathogenic microorganisms to trigger an infection. In addition, 30% of the African strains of *S*. *aureus* isolated from all types of samples have been shown to produce the LPV toxin [24, 25].

In general, hospital bacteria are resistant to several classes of antibiotics. Therefore, beyond the ubiquitous nature of staphylococcal strains, we must add their exceptional ability to develop multidrug resistance to several antibiotics [26]. The *S*. *aureus* strain isolated in this study showed a high level of resistance to cephalothin followed by fosfomycin and cefoxitin. On the other hand, the relatively low resistance rate of *S*. *aureus* isolates was observed with chloramphenicol and vancomycin. This resistance to cephalothin recorded suggests that these strains have already been in contact with this generation of cephalosporin. In addition, this confirms the presence of methicillin-resistant *S*. *aureus* since the cephalothins are only active on sensitive *S*. *aureus*. Similarly, resistance rate to fosfomycin and cefoxitin on clinical strains was observed in Brazzaville [27]. Considering fosfomycin, our results are contrary to those obtained in Algeria on clinically isolated *S*. *aureus* where it was about 90% of sensitivity [28]. This difference observed between our results may be explained by the intensity of the contact between this antibiotic and the *S*. *aureus* strains in these two countries. The resistance rate to cefoxitin (87.5%) observed in our study is higher than the 43% obtained on *S*. *aureus* in the hospital environment at the public hospital center of Boufarik in Algeria [29]. These results suggest that 87.5% of *S*. *aureus* obtained in our study is resistant to methicillin (MRSA). Our recorded data are much higher than the rate of MRSA observed in French hospitals, which was from 10% to 16.5% in 2016 [30]. Nevertheless, *S*. *aureus* showed weak resistance to chloramphenicol (15%) and vancomycin (25%). Indeed, a low resistance rate for chloramphenicol (0.6%) had been mentioned on community-acquired *S*. *aureus* in Morocco [31].

The proportion of resistance to vancomycin is lower than the 63.63% obtained on clinical strains of *S*. *aureus* [27]. However, the efficacy of vancomycin has been demonstrated both on food [32] and clinically isolated *S*. *aureus* strains [29]. In addition, Daurel et al. [33] estimated that approximately 90% of MRSA is hospital-based and that vancomycin may be an alternative for resistance. Therefore, according to observed results, chloramphenicol and vancomycin could be alternative molecules in cases of hospital-acquired MRSA infections. CNS have also high proportions of resistance to fosfomycin and penicillin. Data recorded with fosfomycin are contrary to those published in Algeria [34]. This difference could be explained by a very moderate use of fosfomycin on staphylococcal strains in Algeria. Meanwhile, they reported about 60% resistance of CNS to penicillin. However, the CNS have shown low resistance to gentamycin and ciprofloxacin. This finding on the low rate of resistance to gentamycin and ciprofloxacin had been observed on clinical CNS isolates in Mali [21]. Given all these results, we believe that an improvement in antibiotic therapy must be taken seriously in these various services.

Many staphylococci have the capacity to produce biofilm, which makes it easier for them to adhere to medical equipment and surface. The biofilm formation test reveals that 37% of our isolates were biofilm formers. This rate is lower than the 89% obtained on staphylococcal strains isolated from medical implants in Algeria [35]. Considering species, it is observed that all *S*. *lugdunensis* and *S*. *warneri* isolated were formative of biofilm followed by *S*. *epidermidis* (60%). This proportion of biofilm formation by *S*. *lugdunensis* and *S*. *warneri* is higher than the result obtained by Ahouandjinou [36], which was 28% and 20%, respectively, for *S*. *lugdunensis* and *S*. *warneri* on food *Staphylococcus* spp. strains. This difference could be explained by the low representativeness of these isolates and the origin of collected samples. Our results on *S*. *epidermidis* corroborate those of Kara-Terki [37], who revealed that 53.5% of the strains of *S*. *epidermidis* isolated from urinary catheters were biofilm-forming. However, no biofilm formation was noticed with species such as *S*. *cohnii* ssp. *cohnii*, *S*. *haemolyticus*, and *S*. *saprophyticus* isolated in this study. We can say that isolated *S*. *aureus* and biofilm-forming CNS are dangerous germs since their virulence also resides in the capacity to produce an extracellular matrix and constitute a biofilm [38].

It should be remembered that in our study, the influence of biofilm formation on antibiotics resistance was not observed since some strains, although biofilm-forming, were found to be sensitive to certain antibiotics. This could be explained by the fact that we used planktonic colonies to carry out the susceptibility assay. This is why Fitzpatrick et al. [38] consider that antibiotic usually active on bacteria in the planktonic state often proves to be less effective on structures organized in biofilm. Therefore, the eradication of a bacterial biofilm represents a big clinical problem.

## 5. Conclusion

Among the thirteen staphylococcal species identified in the hospital environment, *S*. *aureus* was the only coagulase-positive staphylococci isolated. These isolates are of various origins, and this implies poor practice of hygienic rules. It is also observed that these identified *Staphylococcus* strains display variable resistance profiles to tested antibiotics. Antibiotics such as chloramphenicol and vancomycin are more effective on *S*. *aureus*. The coagulase-negative *Staphylococcus* strains developed less resistance to gentamycin and ciprofloxacin. The capacity of staphylococcal cells to form biofilm was high with *S*. *lugdunensis*, *S*. *warneri*, and *S*. *epidermidis* strains. Among the prevention strategies, the optimal use of antibiotics is the cornerstone of the reduction of antibiotic resistance. However, regular monitoring of hospital hygiene is crucial with the use of biodetergents suitable for combating the formation of bacterial biofilms. To end, an evaluation of toxin production by isolated species and a molecular characterization could better inform on their pathogenicity level.

## Figures and Tables

**Table 1:** Antibiotic resistance profile of isolated *Staphylococcus* strains.

Antibiotics	*S*. *aureus* resistance rate (%)	CNS resistance rate (%)
Cefoxitin	87.50	66.00
Kanamycin	80.00	42.00
Erythromycin	75.00	54.00
Fosfomycin	92.50	94.00
Vancomycin	25.00	25.00
Cephalothin	100.00	81.00
Trimethoprim	85.00	64.00
Tetracycline	47.50	46.00
Penicillin G	80.00	87.00
Chloramphenicol	15.00	26.00
Streptomycin	30.00	21.00
Amoxiclav	75.00	34.00
Gentamycin	52.50	17.00
Ciprofloxacin	45.00	17.00
Ceftriaxone	80.00	58.00

## Data Availability

The data used to support the findings of this study are available from the corresponding author upon request.
